# Effect of ciprofloxacin in the ultrastructure and development of biofilms formed by rapidly growing mycobacteria

**DOI:** 10.1186/s12866-015-0359-y

**Published:** 2015-02-04

**Authors:** María-Carmen Muñoz-Egea, María García-Pedrazuela, Ignacio Mahillo, Jaime Esteban

**Affiliations:** Department of Clinical Microbiology, IIS- Fundación Jiménez Díaz. Av. Reyes Católicos 2, 28040 Madrid, Spain; Department of Epidemiology, IIS- Fundación Jiménez Díaz Av. Reyes Católicos 2, 28040 Madrid, Spain

**Keywords:** Mycobacteria, Biofilm, Antibiotic, Ciprofloxacin

## Abstract

**Background:**

The aim of this study was to analyze the effect of ciprofloxacin at different times on the development and behavior of intrinsic autofluorescence, covered area, thickness and cell viability in a biofilm formed by non-pigmented rapidly growing mycobacteria (NPRGM).Confocal laser scanning microscopy and image analysis were used to study the behavior of ciprofloxacin on biofilms.

**Results:**

Thickness was the most affected parameter, although some species showed changes in other parameters. At the same time, we also measured the minimum inhibitory concentration and the minimum biofilm eradication concentration (MBEC). An increase in MBEC was observed in all the strains, *M. peregrinum* being the species that presented the highest increase.

**Conclusions:**

This study help us to understand better how mycobacterial biofims can be affected by ciprofloxacin.

## Background

It is now considered that bacteria can have two forms during their growth and proliferation [1]: single independent cells (planktonic cells), and sessile communities of cells surrounded by an extracellular matrix (biofilms) [2]. One of the most important characteristics of biofilm-based infections is the development of a resistance to conventional antimicrobial agents, and to host defenses, that make difficult the management of these infections [3].

Several studies have demonstrated the ability of non-pigmented rapidly growing mycobacteria (NPRGM) to form biofilms [4], and their diminished susceptibility to commonly used antibiotics [5]. Although it is generally agreed that bacteria increases their resistance to antibiotics inside the biofilm, to the best of our knowledge there are no reports about the effect of antibiotics on a biofilm at different stages of development. This knowledge could be of importance, because it can allow developing new strategies for management of these diseases. In this study, we analyze the effect of ciprofloxacin on the growth and the structure of mycobacterial biofilms during their development.

## Material and methods

*Mycobacterium abscessus* DSM 44196, *Mycobacterium chelonae* ATCC 19235, *Mycobacterium fortuitum* ATCC 6841, *Mycobacterium mageritense* ATCC 700351, *Mycobacterium mucogenicum* DSM 44124, *Mycobacterium peregrinum* ATCC 14467, and *Mycobacterium smegmatis* ATCC 607 were used in the experiments.

Biofilm development was analyzed at 24, 48, 72, and 96 h using hydrophobic uncoated sterile slide 2- by 4-well plates (ibidy GmbH, Martinsried, Germany), as follows. Mycobacterial colonies were resuspended in sterile phosphate buffered saline solution (PBS) (bioMérieux, France) to achieve a cell density of 1.5 × 10^8^ CFU/ml. Three hundred microliters of this suspension was inoculated on each well. Inoculated slides were incubated at 37°C in a 5% CO2 atmosphere for 30 min. The suspension was then removed, and the wells were washed once with PBS. All the experiments were done in parallel with and without antibiotic. After bacterial inoculation, 300 μl of Middlebrook 7H9 broth (7H9) (BD, Estados Unidos) was added in each well throughout the experiment, and 300 μl of ciprofloxacin (CIP) (Sigma, Germany) 2 μg/ml (suspension in water solution) at 24, 48 and 72 hours. The antibiotic was maintained until the end of the experiment, leaving one well without antibiotic as a control with 7H9 (96 hours). The slides were placed on an orbital shaker (80 rpm) and incubated at 37°C in normal atmosphere for 4 days. The concentration of CIP (1 μg/ml) was selected following the breakpoints indicated for these organisms in the Clinical and Laboratory Standards Institute (CLSI) guidelines [6] and the concentrations that have been described in humans after IV administration [7]. Slides were examined, and the medium with and without antibiotic was changed daily. The slide wells were stained using Live/Dead BackLight^©^ stain (Invitrogen, Eugene, OR) and Nile Red^©^ stain (Sigma-Aldrich Co., St. Louis, MO). Stains were performed according to the instructions provided by the manufacturer. At the end of the experiment (at 96 hour incubation) and after staining, plates were analyzed using a Leica DM IRB confocal laser-scanning microscope (Leica, Germany). All the experiments were performed in triplicate for each strain.

One set of wells was used per NPRGM species to study both autofluorescence and Nile Red stain, and the other was used to analyze the proportion of live and dead mycobacteria. All materials managed in the experiments emitted no autofluorescence. The covered surface was studied by taking 24 microphotographs for each stain, bacterium, and time set. Photographs were analyzed as previously described [8]. The thickness of the biofilm was measured in eight predefined points per well. Autofluorescence was analyzed as the percentage of fluorescence related to the number of bacterial cells detected with the Nile Red stain using the following formulation: (% autofluorescence of covered surface/% Nile Red covered surface) × 100.

The values of autofluorescence, percentage of covered surface, percentage of deaths, and thickness were measured and compared between the different groups regarding antibiotic administration times (24, 48, 72 hours). Groups were compared by pairs using Mann-Whitney test with a significance level of 0.05. Separate comparisons were performed for each species. EPI-INFO 3.5.1. epidemiological software was used for statistical calculations (Centers for Disease Control and Prevention, Atlanta, GA).

Susceptibility testing of NPRGM was performed following the CLSI guidelines [6]. Minimal Inhibitory Concentrations (MICs) were determined using serial concentrations of CIP (0.125 to 16 mg/L) in sterile P-96 microplates (Corning, USA). Each plate contained two wells for growth control and two wells without bacteria as a sterility control of the medium with Mueller-Hinton II (Difco, Detroit, MI). To determine the Minimum Biofilm Eradication Concentration (MBEC) the biofilm was developed in triplicate following the Calgary system with 96-well plates *MBEC™Biofilm Inoculator* (Innovotech, Canada) and it was used according to instructions from the manufacturer with an increased period of incubation (7 days) [9]. Additionally, 7H9 was employed in the first part (96 hours) and Mueller-Hinton broth was used for antibiotic exposure (48 hours).

## Results

**Figure 1:**
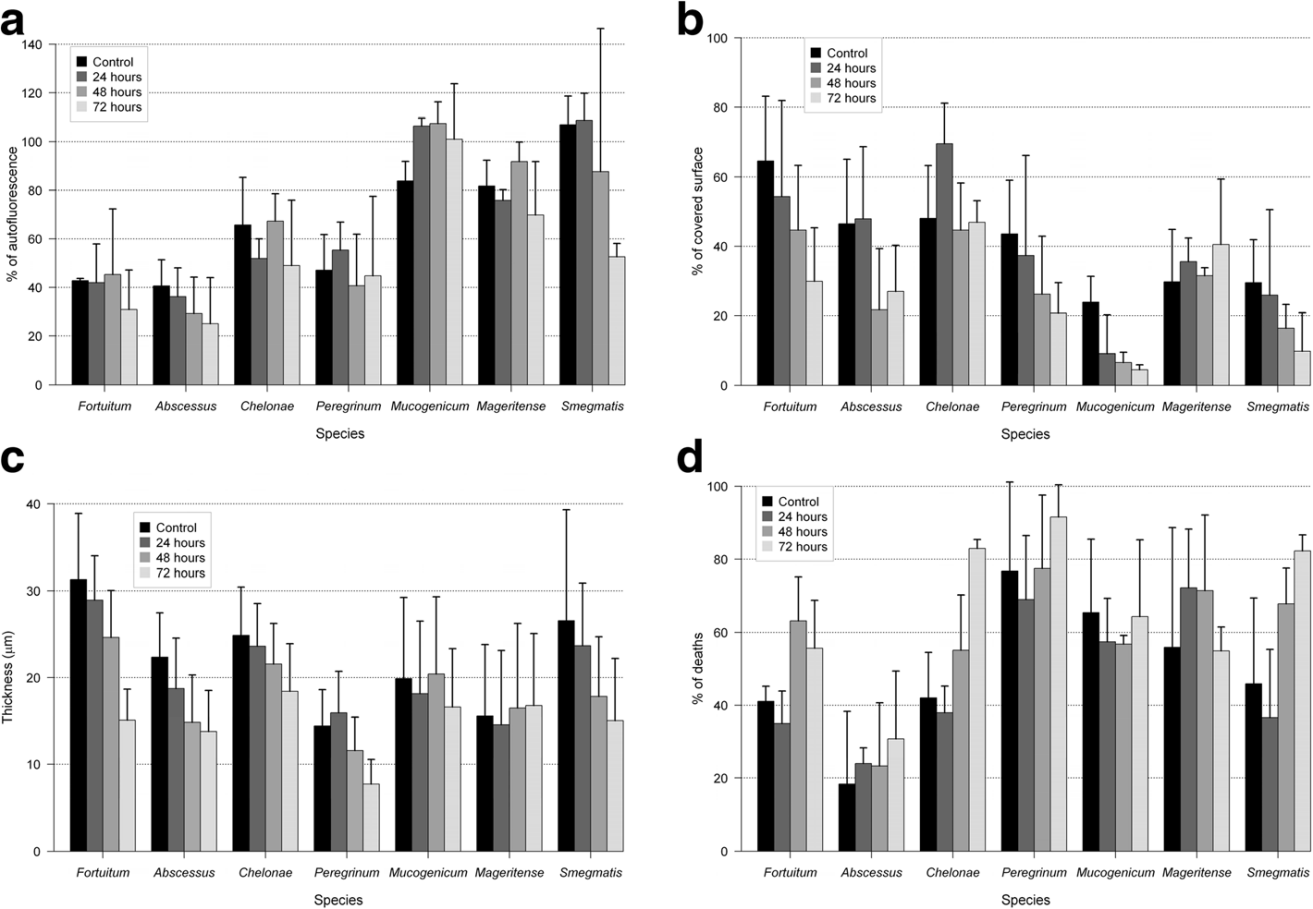
**Effect of ciprofloxacin in the different parameters measured for the biofilm of the different species throughout the experiment. (a)** Percentage of autofluorescence; **(b)** Percentage of covered surface; **(c)** Thickness; **(d)** Percentage of dead bacteria in side the biofilm. Time: hours of incubation with ciprofloxacin (24, 48, 72 h) and control without antibiotic (96 h).

Figure 1 shows the mean and standard deviation results of the different measures for all species. Almost all strains showed statistically significant differences in the thickness measurements between some of the values and the control (all except *M. mageritense*) with p values < 0.005, except for *M. chelonae* (p = 0.04). Regarding % of dead bacteria, differences appeared only in *M. chelonae* and between some measures of *M. fortuitum* and *M. smegmatis. M. chelonae* also showed some differences in the % of covered surface, but these differences cannot be found in other species. Autofluorescence was also affected only in some species (*M. fortuitum, M. mageritense* and *M. smegmatis*). Considering the whole data, it seems that *M. chelonae* and *M. smegmatis* biofilms had more differences in the studied parameters than all other species.

## Discussion

MICs and MBECs are given in Table 1. Comparing MICs and MBECs results, *M. peregrinum* presents the highest increase (>30.000 times), and *M. mucogenicum* the lowest one (>100 times). We have found no relationship between these increases and some of the measurements (especially thickness) of the studied strains. CIP is an active drug for some species but not for others. *M. fortuitum*, *M. smegmatis* and *M. mucogenicum* are typically more susceptible to antibiotics, being normally sensitive to amikacin, cefoxitin, imipenem, ciprofloxacin, sulfonamides and moxifloxacin [10]. Conversely, *M. abscessus* and *M. chelonae* are more resistant to antimicrobials being generally resistant to quinolones and susceptible to amikacin, imipenem and clarithromycin [10]. *M. abcessus* is especially resistant to quinolones, hence a high resistance to CIP and combinations thereof are observed. The resistance of mycobacteria to antibiotics is caused, in part, by its growth rate, hydrophobicity and waterproofness [11,12]. We have previously reported that permeability is not a feature that affects the activity of CIP on NPRGM biofilms [5]. The resistance may be associated to metabolic changes as described previously in *M. abscessus* with clarithromycin, inactive against the stationary-phase state (mature biofilms) [13].

**Table 1:** **Minimum inhibitory concentration (MIC) and minimum biofilm eradication concentration (MBEC) of the different strains against ciprofloxacin**

	***M. abscessus***	***M. fortuitum***	***M. chelonae***	***M. mageritense***	***M. mucogenicum***	***M. peregrinum***	***M. smegmatis***
**MIC (mg/L)**	2	0.06	0.5	0.5	2	0.12	0.25
**MBEC (mg/L)**	1024	256	512	1024	256	4096	512

In this report, we have demonstrated that ciprofloxacin has an important effect on several parameters of biofilms formed by NPRGM, being related to the moment when the antibiotic is added to the growing biofilm. Overall, the thickness of biofilms is the highest affected parameter in most species, but the effect of ciprofloxacin is not so high on the other parameters (Table 2). An explanation for the effect on thickness could be that it may be easier to kill the bacteria of the biofilm external surface because they have an active metabolism that can be more easily affected than other bacteria inside the structure [14]. The fact that metabolically active surface layers of biofilm are more exposed to lethal doses of antibiotic could also explain these results.

**Table 2:** **Mean and standard deviation of the different parameters (thickness, death, covered surface, autofluorescence) of each strain under different conditions of inoculation (24, 48, and 72 hours of exposure to the ciprofloxacin), and control without antibiotic (96 hours)**

	**Thickness (μm)**				**% of deaths**				**% of covered surface**				**% ofautofluorescence**			
	**Control (96 h)**	**24 h**	**48 h**	**72 h**	**Control (96 h)**	**24 h**	**48 h**	**72 h**	**Control (96 h)**	**24 h**	**48 h**	**72 h**	**Control (96 h)**	**24 h**	**48 h**	**72 h**
*M. fortuitum*	31.3 ± 7.6	28.9 ± 5.1	24.6 ± 5.4*	15.1 ± 3.6*	41.1 ± 4.1	35 ± 8.9	63.2 ± 12.1*	55.6 ± 13.2	64.6 ± 18.5	54.3 ± 27.6	44.6 ± 18.7	29.9 ± 15.4	42.8 ± 0.9	42 ± 15.9	45.3 ± 26.9	30.8 ± 16.2
*M. abscessus*	22.4 ± 5.1	18.8 ± 5.8*	14.8 ± 5.4*	13.8 ± 4.7*	18.4 ± 20	24 ± 4.3	23.3 ± 17.4	30.8 ± 18.6	46.5 ± 18.6	47.9 ± 20.8	21.7 ± 17.6	27 ± 13.3	40.6 ± 10.8	36.2 ± 11.8	29.2 ± 15	25 ± 19
*M. chelonae*	24.9 ± 5.6	23.6 ± 4.9	21.6 ± 4.7*	18.4 ± 5.5*	42 ± 12.4	38 ± 7.3	55 ± 15.2	82.9 ± 2.5*	48 ± 15.2	69.6 ± 11.6	44.7 ± 13.6	46.9 ± 6.3	65.7 ± 19.6	51.9 ± 8.1	67.3 ± 11.3	48.9 ± 27
*M. peregrinum*	14.4 ± 4.2	15.9 ± 4.8	11.6 ± 3.9*	7.7 ± 2.8*	76.7 ± 24.4	69.1 ± 17.5	77.4 ± 20.2	91.6 ± 8.8	43.6 ± 15.4	37.4 ± 28.8	26.2 ± 16.7	20.7 ± 8.8	47 ± 14.7	55.3 ± 11.5	40.7 ± 21.2	44.8 ± 32.7
*M.mucogenicum*	19.9 ± 9.3	18.1 ± 8.4	20.4 ± 8.9	16.6 ± 6.7	65.4 ± 20.1	57.4 ± 11.9	56.8 ± 2.4	64.4 ± 20.9	23.9 ± 7.4	9.2 ± 11.1	6.6 ± 2.9*	4.5 ± 1.4*	83.8 ± 8	106.5 ± 3.2*	107.5 ± 8.8*	101 ± 22.7
*M. mageritense*	15.6 ± 8.2	14.5 ± 8.6	16.5 ± 9.7	16.8 ± 8.3	55.9 ± 32.8	72.2 ± 16	71.5 ± 20.7	54.9 ± 6.6	29.7 ± 15.1	35.5 ± 6.8	31.5 ± 2.3	40.5 ± 18.9	81.8 ± 10.6	75.8 ± 4.4	91.8 ± 8	69.8 ± 22
*M. smegmatis*	26.6 ± 12.7	23.7 ± 7.2	17.8 ± 6.9*	15 ± 7.2*	33.2 ± 11.5	36.6 ± 18.7	67.9 ± 9.7	82.3 ± 4.4	26.5 ± 15.8	25.9 ± 24.7	16.4 ± 6.9	9.8 ± 11	101.5 ± 9.5	108.8 ± 11	87.7 ± 58.8	52.6 ± 5.5

*p < 0.05 compared with control.

**Figure 2:**
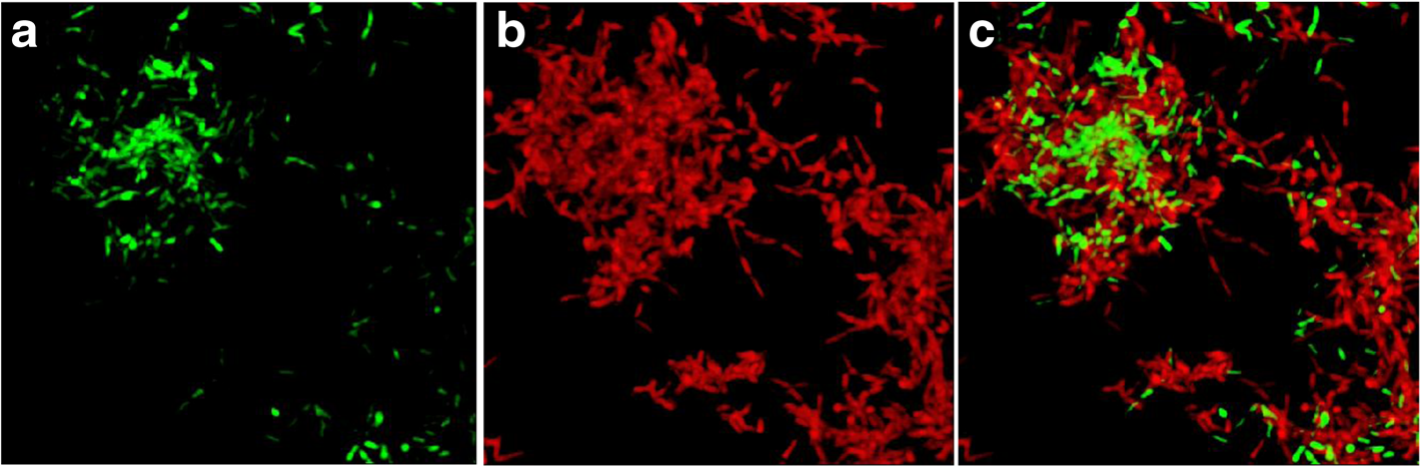
**Live/Dead bacteria in a biofilm formed by** ***M. smegmatis*** **after 72 hours of ciprofloxacin exposure (Live/Dead Backlight** ^**©**^ **stain) using a Lecia DM IRB confocal laser-scanning microscope. (a)** Live bacteria; **(b)** Dead bacteria; **(c)** Live/Dead bacteria.

When compared to the controls, there is a remarkable reduction to 1/2 dead bacteria after 72 h in *M. chelonae* and *M. smegmatis* (Figures 1 and 2). We also found that the wells treated 24 h presented an average percentage of dead bacteria a little bit lower than controls. In this case, differentiation of bacterial cells that can lead to resistance could have appeared in early stages of biofilm development [13], but the exposure time could have also been of importance. Nevertheless, we can find live bacteria even in a 24 hour-developed biofilm and after a 72-hour exposure to antibiotics. This fact can imply that clinical resistance can appear even in the pre-symptomatic steps of disease.

Regarding covered surface, it is interesting that no statistically significant differences between controls and those treated 24 hours with ciprofloxacin were found. Perhaps during the first steps of a biofilm formation it is more difficult to detach cells from the surface [15]. Interestingly, the percentage of covered surface is higher in *M. fortuitum*, *M. chelonae* and *M. abscessus*, the most clinically relevant species of this group [16].

**Figure 3:**
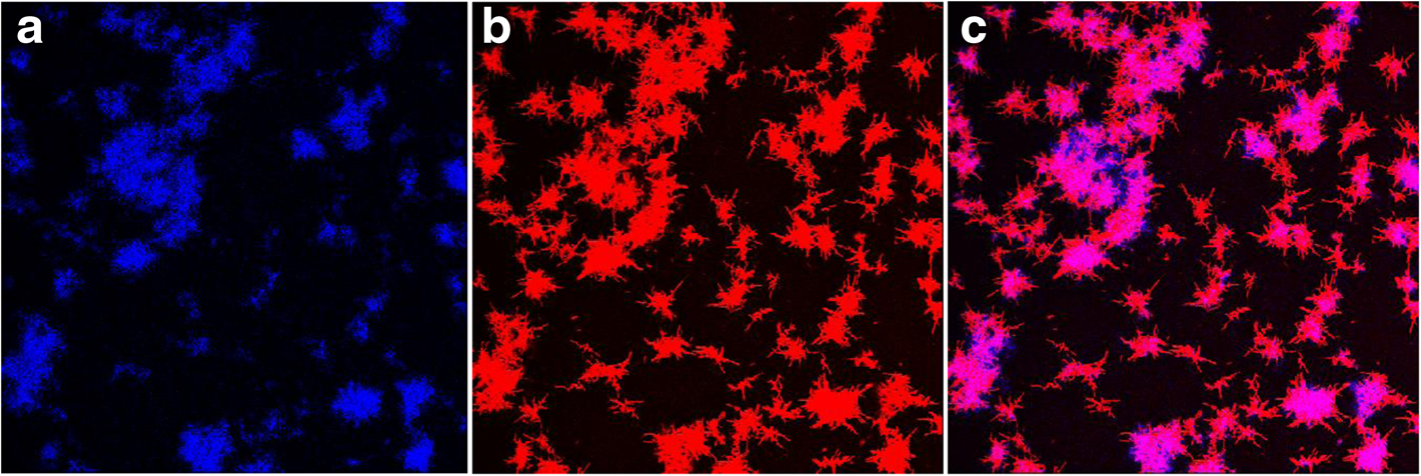
**Extracellular autofluorescence in a biofilm formed by** ***M. smegmatis*** **after 72 hours of ciprofloxacin exposure using a Leica DM IRD confocal laser-scanning microscope. (a)** Autofluorescence; **(b)** Bacteria stained with Nile Red^©^; **(c)** Images a+b.

The presence of autofluorescence seems not to be grossly affected by ciprofloxacin, except in the case of *M. smegmatis*, where after 72 hours of exposure to ciprofloxacin the percentage of autofluorescence is reduced to almost 1/2 (Figure 1). Moreover, in *M. mucogenicum* and *M. smegmatis* we detected the presence of extracellular autofluorescence (Figure 3). In a previous study, we have demonstrated this property outside the bacterial cells, probably in the extracellular matrix, and even outside the biofilm [17]. The causes of the autofluorescence remain speculative, but it can be affected by antibiotic treatment in some of the studied species.

The main limitation of the study is related to the fact that only some aspects of biofilm development were studied. Further studies, including the study of other antibiotics against these species, and also the analysis of molecular aspects in biofilms (like gene activation, metabolism, etc.), are necessary to better understand the behavior of biofilms formed by NPRGM under the effect of antibiotics.

## Conclusions

According to our results it can be concluded that when rapidly growing mycobacteria are part of a biofilm they have a much higher resistance to antimicrobials compared to planktonic mycobacteria. CIP is an active drug for some species of NPRGM and it has been probed that this antibiotic has an important effect on several parameters of biofilms formed by NPRGM especially in the thickness.

Little is known about the complexity of the mycobacterial biofilm structure [18], but recent studies showed that the characteristics of these structures are different from those formed by other organisms [19]. Further studies are necessary to continue evaluating the effects caused by different antibiotics, aiming to elucidate how biofims can be affected by them.

## Abbreviations

NPRGM: Non-Pigmented Rapidly Growing Mycobacteria
7H9: Middlebrook 7H9 broth
CLSI: Clinical and Laboratory Standards Institute
PBS: Phosphate Buffered Saline solution
CIP: Ciprofloxacin
MBEC: Minimum Biofilm Eradication Concentration
MIC: Minimal Inhibitory Concentration
